# Knowledge, attitude, and practice of evidence-based medicine among resident physicians in hospitals of Syria: a cross-sectional study

**DOI:** 10.1186/s12909-022-03840-7

**Published:** 2022-11-14

**Authors:** Muhammad Nour Alabdullah, Hadi Alabdullah, Sondos Kamel

**Affiliations:** 1 Otorhinolaryngology Department, Al-Mowassat University Hospital, Damascus University, Damascus, Syrian Arab Republic; 2 Faculty of Medicine, Hama University, Hama, Syrian Arab Republic; 3 Faculty of Civil Engineering, Hama University, Hama, Syrian Arab Republic

**Keywords:** Knowledge, Attitude, Practice, Evidence-based medicine, Residents, Syria

## Abstract

**Background:**

Evidence-based medicine (EBM) is to integrate the best research evidence with our clinical expertise, circumstances, and unique values of our patient. However, there are no studies about using EBM in clinical practice among resident doctors in Syria. In this study, we aimed to evaluate the self-reported knowledge, attitude and practice (KAP) of EBM by resident doctors throughout different teaching hospitals in Syria.

**Methods:**

The study is a cross-sectional. A self-reported online questionnaire was used to collect data about KAP of EBM from 214 resident physicians working in secondary and tertiary teaching hospitals. The study was conducted between September 2021 and February 2022. All data were analyzed using SPSS, and non-parametric statistical tests were used to identify the correlation between different variables and make the necessary comparisons.

**Results:**

Two hundred and fourteen physicians responded to the questionnaire with a response rate of 85.6%. The overall mean scores of KAP of EBM were 59.2, 74.3 and 53.9%, respectively. The participants displayed a low level of awareness of resources and statistical terms used in EBM. The most well-known resources for residents were Up To Date and PubMed. Among the participants, pediatric residents achieved the highest score in practicing EBM, while family medicine residents scored the lowest score.

**Conclusion:**

The overall impression about the KAP of EBM among Syrian residents was as following: weak awareness, neutral attitude and poor practice of EBM. Training workshops should be set up to teach residents the skills needed to move from opinion-based practice to evidence-based practice.

**Supplementary Information:**

The online version contains supplementary material available at 10.1186/s12909-022-03840-7.

## Introduction

Evidence-based medicine (EBM) is to integrate the best research evidence with our clinical expertise, circumstances, and unique values of our patient [1]. Dr. David Sackett provided a definition of EBM as “the conscientious, explicit, and judicious use of current best evidence in making decisions about the care of the individual patient”. It means integrating individual clinical expertise with the best available external clinical evidence from systematic research [2]. This helps in providing quality care by using credible evidence in the clinical decision-making process [3]. The term EBM is firstly used in 1992 and became the way to find the best available health care [4]. Systematic reviews with and without meta-analysis conducted by The Cochrane Library represent the gold standard in practicing of EBM [5]. Applying the EBM in medical practice can improve the quality of provided healthcare especially in developing countries with lack of resources [6]. Evidence-based practice has become used in many healthcare specialties, including dentistry, nursing, physiotherapy, occupational therapy, complementary medicine, and many others. However, evidence-based practice is not limited to healthcare disciplines, but to other fields including education, justice, and policymaking [7, 8]. The knowledge and attitude of healthcare providers toward EBM have an essential effect on their practice of EBM [9]. Previous studies supported the importance of EBM [10–14]. According to Moosavi et al., applying the EBM in the health system is useful for both medical staff and patients, because identifying staff weaknesses in this area can guide policymakers to choose appropriate policies needed in the future [10]. Validated and updated information in therapeutic and diagnostic aspects have undergone rapid growth due to EBM, leading to improve the quality of health care and physicians’ skills and knowledge [11]. Several studies demonstrated considered gaps between scientific evidence and the care provided by healthcare workers [14]. Many studies have been conducted to evaluate the knowledge, attitude, and practice (KAP) of EBM [15–17]. Adeodu et al., studied the attitudes of occupational physicians in the UK towards evidence-based guidelines and found that the physicians showed positive attitude to evidence-based guidelines [15]. A study among Norwegian physicians revealed a limited knowledge of the key aspects of EBM but a positive attitude towards the concept. They were indifferent to the impact of EBM on medical practice and showed limited experience in the practice of EBM [16]. In a systematic review, the majority of participants revealed a positive attitude toward the applying of EBM, although many of them showed poor EBM knowledge and skills [17]. Several studies found that physicians and medical students are interesting in teaching and practicing the EBM [18–22]. In a study conducted in one area of Saudi Arabia, physicians showed welcomed attitude, but limited awareness and practice of EBM [23]. Similar results have been found in a study conducted in Iran [24]. However, no data are available for adoption of EBM by physicians in hospitals of Syria. In this study, we aimed to evaluate the KAP of EBM among residents of various teaching hospitals in Syria.

## Methods

### Study design

A cross-sectional study was performed between September 2021 and February 2022. A self-reported online questionnaire created using Google Form was used to collect data about KAP of EBM from resident physicians working in secondary and tertiary teaching hospitals in Syria. The study included the residents in the five largest teaching hospitals in Damascus and Aleppo governorates (Almowassat University Hospital, Alassad University Hospital, Almoujtahed Hospital, Alrazi Hospital, and Tishreen Military Hospital).

### Sampling

The sample size was calculated using Raosoft online software available at “http://www.raosoft.com/samplesize.html”. The number of resident doctors in the aforementioned hospitals is about 5000. Based on that, assuming a confidence level of 95%, a response distribution of 50%, and a margin of error of 7%, the required sample size was 189. Stratified random sampling was used to select 250 participants from the five mentioned hospitals. Inclusion criteria included that the resident is: (1) Spent at least 6 months of residency, (2) has an access to the electronic mails, (3) willing to participate and complete the questionnaire. All those who did not complete the first 6 months of residency was not included.

### Study tool

The used questionnaire was consisted of seven sections and was translated into Arabic with the assistance of Arabic language specialists, therefore, it was formulated clearly and conceptually (English version in the Additional file 1). The questionnaire was piloted on 15 participants from different hospitals and specializations and some adjustments were made based on their feedback to confirm that all participants can understand and answer the required questions. The first two sections included informed consent and socio-demographic characteristics of residents. Sections from 3 to 5 contained validated Noor EBM questionnaire which includes 15, 17, and 11 statements to define KAP of EBM, respectively [9]. Regarding to the domains of the Noor EBM questionnaire, we used a five-point Likert scale using the Strongly Agree = 5/Agree = 4/Neutral = 3/Disagree = 2/Strongly Disagree = 1 scale for the knowledge and attitude domains, and using the Always = 5/Often = 4/Sometimes = 3/Seldom = 2/Never = 1 scale for the practice domain. Reverse scoring was used for negatively worded items. We calculated total points for each domain. The total score for each row was transformed into percent score by dividing it with the maximum score and multiplying it by 100. According to Bloom’s cut-off point, percent scores which are within the range (60–79%) are defined as moderate knowledge, neutral attitude, and fair level of practice of EBM. Scores above this range are considered as a high level of knowledge, positive attitude, and a good level of practice. In contrast, Scores below this range are defined as low knowledge, negative attitude, and poor practice [25]. Sections 6 and 7 evaluate participants’ knowledge and use of some of the most common resources and statistical terms used in EBM. We used internal consistency reliability-based Cronbach’s alpha statistic to estimate the questionnaire’s construct validity and reliability. A Cronbach’s alpha coefficient ≥ 0.7 was considered acceptable.

### Data collection

The names of all residents with their email addresses have been obtained in each hospital separately. The questionnaire was sent to each participant through an email. The participants were informed of the purpose of the study and were invited to provide consent to participate at the outset of the survey, therefore, participation was voluntary and anonymous. A week later, a reminder was sent to the physicians who had not respond for the first time.

### Data analysis

All collected data was extracted from Google Form directly to an Excel spreadsheet and appropriately coded in order to make it applicable to statistical tests. All data were analyzed using IBM SPSS Statistics version 26. The descriptive statistics were calculated, including mean values, standard deviations, sum scores, frequencies, and percentages of the relevant factors. As the data had asymmetrical distribution, we decided to use non-parametric statistical tests to identify the correlation between different variables and make the necessary comparisons; Mann–Whitney U test was applied for variables with 2 categories and Kruskal-Wallis test was used for those with 3 or more categories. Logistic Regression was conducted to study the correlation between some variables. A *p* value of < 0.05 was considered statistically significant.

### Ethical consideration

The research was approved by the Research Ethics Committee, Faculty of Medicine, Damascus University with approval code (no. 2178/2021). Informed consent was obtained from each individual prior to participation.

## Results

Of 250 participants, 214 physicians agreed to participate and fully completed the questionnaire (response rate of 85.6%). The participants included 119 males (55.6%). The average age of the participants was 27.7 years (Std. Deviation 2.3; range 24–33 years). The average year of residency was 3.8 years (Std. Deviation 1.7; range 1–7 years). Of the participants, 82 residents (38.3%) were training at Ministry of Higher Education hospitals, 65 residents (30.4%) were training at Ministry of Health hospitals and 67 residents (31.3%) were training at Ministry of Defense hospitals. Socio-demographic characteristics of participants are shown in Table 1. The number of residents who had previous training in EBM was 48 residents (22.4%). The overall Cronbach’s alpha scores for the KAP sections were 0.7, 0.8 and 0.7, respectively. Therefore, the used questionnaire is validated and reliable. The mean total scores for the KAP of EBM domains were 44.4 (59.2%), 63.1 (74.3%) and 29.7 (53.9%), respectively (Table 2).

**Table 1:** Participants’ socio-demographic characteristics

*Variable*	*n (%), unless otherwise* *specified*
Age (mean ± Std. deviation)	27.7 ± 2.3
Gender	
Male	119 (55.6)
Female	95 (44.4)
Specialty	
Surgery	26 (12.1)
Medicine	20 (9.3)
Pediatric	22 (10.3)
Obstetrics and Gynecology	19 (8.9)
Ophthalmology	14 (6.5)
Otolaryngology	17 (7.9)
Dermatology	14 (6.5)
Family medicine	23 (10.7)
Oncology	16 (7.5)
Others	43 (20)
Ministry of specialty	
Ministry of Higher Education	82 (38.3)
Ministry of Health	65 (30.4)
Ministry of Defense	67 (31.3)
Year of residency	
1th	15 (7)
2th	44 (20.6)
3th	40 (18.7)
4th	41 (19.2)
5th	33 (15.4)
6th	25 (11.7)
7th	16 (7.5)
Previously undergone any training courses in EBM	
Yes	48 (22.4)
No	166 (77.6)

**Table 2:** Mean total scores for KAP of EBM according to the Noor EBM questionnaire

	Total score (n/N)	Mean score (%)	Std. deviation	General impression
Knowledge	9497/16050	44.4 (59.2)	8.5	Low knowledge
Attitude	13,514/18190	63.1 (74.3)	10.4	Neutral attitude
Practice	6346/11770	29.7 (53.9)	7.4	Poor practice

### Knowledge of EBM

The mean score for individual statements of the knowledge domain ranged from 2.5 to 3.4 (Std. Deviation range 1.1–1.3). Over half of the participants (*n* = 118; 55.1%) had a low level of knowledge followed by moderate level (*n* = 75; 35%). However, a limited number of residents (*n* = 21; 9.8%) showed a high level of knowledge. Table 3 shows descriptive statistics of the level of participants’ knowledge of EBM, from which we find that the highest level was awarded to the statement 15: (Application of evidence-based practice is cost-effective to the healthcare system), with mean 3.4 and Std. Deviation 1.3 followed by the statement 2: (Evidence-based medicine focuses on the best currently available research without considering clinical experience), with mean 3.3 and Std. Deviation 1.4, with strongly agree by percent (26.6 and 13.1%, respectively) and agree by percent (23.8 and 18.2%, respectively). While the lower level was awarded to the statement 13: (The increasing number of systematic reviews that are applicable to general practice can be found in the Cochrane Library) with mean 2.8 and Std. Deviation 1.2 followed by the statement 4: (Patients’ preferences should be prioritized over clinicians’ preferences in making clinical decisions) with mean 2.5 and Std. Deviation 1.1 with strongly agree by percent (14 and 15.9%, respectively) and agree by percent (18.7 and 43.9%, respectively).

**Table 3:** Descriptive statistics of participants’ knowledge of EBM

Item	Strongly agree n (%)	Agree n (%)	Neutral n (%)	Disagree n (%)	Strongly disagree n (%)	Mean	Std. Deviation	Level
K1	Evidence-based medicine involves the process of critically appraising research findings as to the basis for clinical decisions.	32 (15)	41 (19.2)	68 (31.8)	37 (17.3)	2.8	1.3	13
K2	Evidence-based medicine focuses on the best current available research without considering clinical experience.	39 (18.2)	40 (18.7)	52 (24.3)	55 (25.7)	3.3	1.4	2
K3	Evidence-based medicine is suitable for making decisions about the care of patients rather than for policymaking.	36 (16.8)	77 (36)	35 (16.4)	30 (14)	2.9	1.3	8
K4	Patients’ preferences should be prioritized over clinicians’ preferences in making clinical decisions.	94 (43.9)	38 (17.8)	33 (15.4)	15 (7)	2.5	1.1	15
K5	Evidence-based medicine improves clinical management by using evidence from meta-analysis only.	46 (21.5)	66 (30.8)	53 (24.8)	22 (10.3)	3	1.2	4
K6	Evidence-based medicine does not help to promote self -directed learning.	48 (22.4)	41 (19.2)	86 (40.2)	13 (6.1)	3.1	1.2	3
K7	Meta-analysis is superior to case-control studies in evidence-based medicine.	26 (12.1)	46 (21.5)	74 (34.6)	23 (10.7)	3	1.3	6
K8	Four essential components structured in the PICO format (Patient or problem, Intervention, Comparison, Outcome) will make a good clinical question.	28 (13.1)	48 (22.4)	54 (25.2)	42 (19.6)	2.9	1.4	11
K9	Evidence-based medicine improves clinicians’ understanding of research methodology.	24 (11.2)	41 (19.2)	69 (32.2)	36 (16.8)	2.9	1.4	12
K10	Clinicians who practice evidence-based medicine become less critical in using data in systemic reviews.	41 (19.2)	66 (30.8)	46 4 (21.5)	28 (13.1)	3	1.2	7
K11	Evidence-based medicine can be practiced in situations where there is doubt about any aspect of clinical management.	53 (24.8)	47 (22)	75 (35)	20 (9.3)	2.9	1.1	10
K12	Improving access to summaries of evidence is appropriate to encourage evidence-based practice.	41 (19.2)	64 (29.9)	31 (14.5)	45 (21)	3	1.3	9
K13	The increasing number of systematic reviews that are applicable to general practice can be found in the Cochrane Library.	24 (11.2)	56 (26.2)	78 (36.4)	26 (12.1)	2.8	1.2	14
K14	Difficulty in understanding statistical terms is the major setback in applying evidence-based medicine.	40 (18.7)	23 (10.7)	54 (25.2)	46 (21.5)	3	1.5	5
K15	Application of evidence-based practice is cost-effective to the healthcare system.	51 (23.8)	50 (23.4)	40 (18.7)	16 (7.5)	3.4	1.3	1

### Attitude towards EBM

The mean score for individual statements of the attitude domain ranged from 2.4 to 4.3 (Std. Deviation range 1.3–1.2). Eighty-five of the participants (39.7%) revealed positive attitude towards EBM and 94 of the participants (43.9%) had neural attitude, while only 35 of the participants (16.6%) showed negative attitude. Table 4 reveals descriptive statistics of the level of participants’ attitude towards EBM. We find that the highest mean is found in statement 16: (I am interested in receiving educational materials on evidence-based medicine as they relate to various topics) with mean 4.3 and Std. Deviation 1.2, followed by statement 17: (I think that educational interventions and incorporating formal teaching of evidence-based medicine at medical education are very important) with mean 4.2 and Std. Deviation 1.0 with strongly agree by percent (61.7 and 50%, respectively) and agree by percent (22 and 29%, respectively). However, the lowest mean is found in statement 7: (I believe that years of clinical experience is more valuable than evidence-based medicine) with mean 3.1 and Std. Deviation 1.6, followed by statement 10: (I am certain that understanding the basic mechanisms of disease is sufficient for good clinical practice) with mean 2.4 and Std. Deviation 1.3 with strongly agree by percent (22.4 and 29.9%, respectively) and agree by percent (23.4 and 28.5%, respectively).

**Table 4:** Descriptive statistics of participants’ attitude of EBM

Item	Description	Strongly agree n (%)	Agree n (%)	Neutral n (%)	Disagree n (%)	Strongly disagree n (%)	Mean	Std. Deviation	Level
A1	I believe that evidence-based medicine is a threat to good clinical practice.	16 (7.5)	28 (13.1)	28 (13.1)	51 (23.8)	91 (42.5)	3.8	1.3	9
A2	I believe practicing evidence-based medicine can improve patient health outcome.	91 (42.5)	64 (29.9)	22 (10.3)	22 (10.3)	15 (7)	3.9	1.3	6
A3	I am keen to learn evidence-based medicine if given the opportunity.	93 (43.5)	61 (28.5)	23 (10.7)	18 (8.4)	19 (8.9)	3.9	1.3	7
A4	I am ready to practice evidence-based medicine in my work.	66 (30.8)	59 (27.6)	31 (14.5)	36 (16.8)	22 (10.3)	3.5	1.4	14
A5	I feel that research findings are very important in my day-to-day management of patients.	67 (31.3)	53 (24.8)	44 (20.6)	37 (17.3)	13 (6.1)	3.6	1.3	13
A6	I feel that evidence-based medicine is of limited value in general practice because management in primary care requires less scientific evidence.	21 (9.8)	22 (10.3)	33 (15.4)	67 (31.3)	71 (33.2)	3.7	1.3	11
A7	I believe that years of clinical experience is more valuable than evidence-based medicine.	48 (22.4)	50 (23.4)	11 (5.1)	39 (18.2)	66 (30.8)	3.1	1.6	16
A8	I am convinced that applying evidence-based medicine in clinical practice increases the effectiveness of my work.	83 (38.8)	71 (33.2)	25 (11.7)	18 (8.4)	17 (7.9)	3.9	1.2	8
A9	I feel confident managing patients with evidence-based medicine.	73 (34.1)	69 (32.2)	41 (19.2)	16 (7.5)	15 (7)	3.8	1.2	10
A10	I am certain that understanding the basic mechanisms of disease is sufficient for good clinical practice.	64 (29.9)	61 (28.5)	42 (19.6)	24 (11.2)	23 (10.7)	2.4	1.3	17
A11	I feel that access to databases is vital in obtaining journals on evidence-based medicine.	85 (39.7)	44 (20.6)	34 (15.9)	18 (8.4)	33 (15.4)	3.6	1.5	12
A12	I feel that reading the conclusions of a systematic review is adequate for clinical practice.	29 (13.6)	31 (14.5)	70 (32.7)	24 (11.2)	60 (28)	3.3	1.4	15
A13	I feel that practicing evidence-based medicine would produce better health practitioners.	122 (57)	39 (18.2)	31 (14.5)	11 (5.1)	11 (5.1)	4.2	1.2	3
A14	I often feel burdened whenever needing to use evidence-based medicine in practice.	15 (7)	16 (7.5)	36 (16.8)	50 (23.4)	97 (45.3)	3.9	1.2	5
A15	I think it is mandatory for physicians to continuously update their knowledge to deliver efficient patient care.	102 (47.7)	62 (29)	32 (15)	11 (5.1)	7 (3.3)	4.1	1.1	4
A16	I am interested in receiving educational materials on evidence-based medicine as they relate to various topics.	132 (61.7)	47 (22)	16 (7.5)	3 (1.4)	16 (7.5)	4.3	1.2	1
A17	I think that educational interventions and incorporating formal teaching of evidence-based medicine at medical education are very important.	107 (50)	62 (29)	27 (12.6)	12 (5.6)	6 (2.8)	4.2	1.0	2

### Practice of EBM

The mean score for individual statements of the practice domain ranged from 2.1 to 3.7 (Std. Deviation range 1.4–1.1). Eighteen respondents (8.4%) were classified as having good level of practice. However, most residents had poor (*n* = 146; 68.2%) followed by fair (*n* = 50; 23.4%) level of practice. Table 5 shows descriptive statistics of participants’ practice of evidence-based medicine. The statement 9: (I share my knowledge of evidence-based medicine with my colleagues) had the highest score with mean 3.7 and Std. Deviation 1.1, followed by statement 1: (I apply evidence-based medicine in practice) with mean 3.3 and standard deviation 1.2 with always choice by percent (26.6 and 26.2%, respectively) and often choice by percent (36.4 and 12.1%, respectively).

**Table 5:** Descriptive statistics of participants’ practice of EBM

Item	Description	Always n (%)	Often n (%)	Sometimes n (%)	Seldom n (%)	Never n (%)	Mean	Std. Deviation	Level
P1	I apply evidence-based medicine in practice.	56 (26.2)	26 (12.1)	71 (33.2)	51 (23.8)	10 (4.7)	3.3	1.2	2
P2	I use multiple search engines for systematic review.	25 (11.7)	22 (10.3)	14 (6.5)	37 (17.3)	116 (54.2)	2.1	1.4	11
P3	I search for evidence-based medicine material from published journals only.	23 (10.7)	23 (10.7)	46 (21.5)	81 (37.9)	41 (19.2)	2.6	1.2	8
P4	I do not have enough time to study evidence-based medicine.	68 (31.8)	61 (28.5)	31 (14.5)	30 (14)	24 (11.2)	2.4	1.4	10
P5	I cannot practice evidence-based medicine due to limitations of the management that I can offer to patients in clinic settings.	35 (16.4)	96 (44.9)	19 (8.9)	41 (19.2)	23 (10.7)	2.6	1.3	4
P6	I use evidence based-medicine for answering the questions in a clinical setting.	16 (7.5)	19 (8.9)	67 (31.3)	65 (30.4)	47 (22)	2.5	1.1	9
P7	I join continuous medical education for an update regarding evidence-based medicine.	33 (15.4)	28 (13.1)	15 (7)	93 (43.5)	45 (21)	2.6	1.4	6
P8	I promote evidence-based practice to my colleagues at the workplace.	39 (18.2)	22 (10.3)	49 (22.9)	34 (15.9)	70 (32.7)	2.7	1.5	3
P9	I share my knowledge of evidence-based medicine with my colleagues.	57 (26.6)	78 (36.4)	52 (24.3)	16 (7.5)	11 (5.1)	3.7	1.1	1
P10	I am involved in the development of clinical practice guideline.	34 (15.9)	24 (11.2)	29 (13.6)	75 (35)	52 (24.3)	2.6	1.4	5
P11	I usually translate a clinical question into a form that can be answered from the literature.	30 (14)	33 (15.4)	35 (16.4)	49 (22.9)	67 (31.3)	2.6	1.4	7

### Statistical terms and EMB resources

The majority of residents showed overall low understanding of all statistical terms used in the survey. Table 6 summarizes the details of participants’ responses toward some of the most used statistical terms, from which we find a significant number of participants (107 (50%), 94 (43.9%), 108 (50.5%), 116 (54.2%), 110 (51.4%), 113 (52.8%), 144 (67.3%), 125 (58.4%), 122 (57%) and 129 (60.3%) showed a lack of awareness but a willing to learn the concept of absolute risk, systematic review, odds ratio, meta-analysis, clinical effectiveness, number needed to treat, confidence interval and heterogeneity, respectively. The overall awareness of the most used EBM resources was poor (Table 7). Most of the participants displayed unawareness of Bandolier, Clinical evidence, Best practice and Medicine (71.5, 61.2, 57.9 and 48.6%, respectively), While 95 residents (44.4%) were aware but not used of Cochrane database. However, the most well-known resource for residents was Up To Date (56.1% read; 24.3% used in clinical decision-making) followed by PubMed (48.1% read; 24.8% used in clinical decision-making). The correlation between different variables and the level of practicing EBM was studied. No statistically significant association was found between practicing EBM and the following variables: gender (Mann Whitney U test *P* = 0.9), year of residency (Kruskal Wallis’ test *P* = 0.1) and ministry of specialty (Kruskal Wallis’ test *P* = 0.05). However, a significant correlation was found between practicing of EBM and each of specialty and previous training (Mann Whitney U test *P* < 0.0001). However, Logistic Regression revealed that the previous training in EBM is significantly correlated with practicing of EBM (*P* < 0.0001). Among the specialties, pediatric residents performed the best followed by dermatology residents, while the performance of family medicine residents was the worst.

**Table 6:** Participants’ awareness of statistical terms

Statistical Term	It would not be helpful to me to understand n (%)	I don’t understand it but wants to n (%)	Some understanding n (%)	Yes, understood and I could explain to others n (%)
Relative risk	46 (21.5)	107 (50)	36 (16.8)	25 (11.7)
Absolute risk	62 (29)	94 (43.9)	28 (13.1)	30 (14)
Systematic review	58 (27.1)	108 (50.5)	26 (12.1)	22 (10.3)
Odds ratio	49 (22.9)	116 (54.2)	35 (16.4)	14 (6.5)
Meta-analysis	52 (24.3)	110 (51.4)	42 (19.6)	10 (4.7)
Clinical effectiveness	25 (11.7)	113 (52.8)	37 (17.3)	39 (18.2)
Number needed to treat	33 (15.4)	144 (67.3)	19 (8.9)	18 (8.4)
Confidence interval	21 (9.8)	125 (58.4)	26 (12.1)	42 (19.6)
Heterogeneity	42 (19.6)	122 (57)	39 (18.2)	11 (5.1)
Publication bias	45 (21)	129 (60.3)	31 (14.5)	9 (4.2)
*Mean*	43.3 (20.2)	116.8 (54.6)	31.9 (14.9)	22 (10.3)

**Table 7:** Participants’ awareness of EBM resources

EBM resources	Unaware n (%)	Aware but not used n (%)	Read n (%)	Used to help in clinical decision-making n (%)
Bandolier	153 (71.5)	33 (15.4)	17 (7.9)	11 (5.1)
Clinical evidence	131 (61.2)	30 (14)	36 (16.8)	17 (7.9)
Cochrane database of Systematic Reviews	70 (32.7)	95 (44.4)	30 (14)	19 (8.9)
Best practice	124 (57.9)	63 (29.4)	20 (9.3)	7 (3.3)
PubMed	32 (15)	26 (12.1)	103 (48.1)	53 (24.8)
Up To Date	15 (7)	27 (12.6)	120 (56.1)	52 (24.3)
Google scholar	39 (18.2)	82 (38.3)	61 (28.5)	32 (15)
Medicine	104 (48.6)	41 (19.2)	47 (22)	22 (10.3)

## Discussion

Internationally, many studies have focused on EBM among health-care providers. To the best of our knowledge, no studies about KAP of EBM among residents or practicing physicians have been conducted in Syria. However, only one paper studied the EBM among undergraduate medical students in Syria [14]. Syria is a country devastated by over 10 years of war. The quality of medical services provided in hospitals of Syria has declined dramatically during and after the political crisis. In many of developing countries, the cost of treatment shoulders by the poor patient himself or his family. Therefore, it could be frustrating to prescribe a lot of expensive and unsuitable tests or treatments for the patient. However, practicing EBM in developing countries could save a lot of money to use in other aspects of people’s lives. Evidence-based medicine is a scientific advancement that improves clinical settings and leads to better, safer and more cost-effective clinical practice [10]. Norhayati et al. conducted a study about the validity and reliability of the Noor EBM questionnaire and concluded that the questionnaire can be used as a reliable and validated tool to evaluate the KAP of EBM by healthcare professionals [9]. The response rate of our study was 85.6%, which is considered a good response rate compared to other studies [11–13, 23]. Regarding the knowledge pillar, a limited number of participants showed high level of knowledge, while the majority had low level. In comparison with a study performed among emergency doctors in Kelantan, Malaysia, it was found that 49.7% of the participants had a high level of knowledge, followed by moderate (47.5%) and low (2,8%) levels of knowledge [13]. The majority of Syrian residents had a neutral attitude toward EBM. This is unlike to the results of other international studies which showed welcoming attitude toward EBM [11, 13, 23]. The poor level of participants’ knowledge could be the reason of wavering attitude toward the EBM. However, more than 70% (*n* = 155) of residents believed that practicing EBM can improve patient health outcome. Again, over 70% (*n* = 154) of residents were keen to learn EBM if given the opportunity. Regarding the practice of EBM, a limited number of residents had a good level of practice, while the majority showed poor level. Such results are consistent with the low levels of knowledge and attitude shown by the participants. Similar results shown by many studies conducted in other regions [26]. The limited practice of EBM could be the result of a range of obstacles such as a lack of time, a lack of sources published in the native language and insufficient skills required in evidence-based practice [27]. Awareness of statistical terms is essential in the process of critically appraising research findings as to the basis for clinical decisions. The participants showed poor overall understanding of the most common statistical terms used in EBM. However, they expressed interest in learning such terms. The overall awareness of the most popular EBM resources was low for the majority of residents. This could be due to lack of training programs and limited access to EBM resources. Participants who attended previous training courses achieved better results in practicing EBM. Several methods of teaching EBM were reported in the literature. These methods include morning reports, workshops, journal clubs and teaching principles of EBM through the curriculum of medical schools [28–31]. The main limitation of this study is the residents’ self-assessment of their survey, and in this situation they may provide erroneous assessment of different statements included in the questionnaire. However, further studies are required to determine barriers impede evidence-based practice. Thus, we could suggest some solutions to move from opinion-based practice to evidence-based practice.

## Conclusion

Based on the participants’ responses to the questionnaire, the general impression about the EBM among Syrian residents was as following: low knowledge, neutral attitude and poor practice. However, the resident doctors revealed a low level of awareness of resources and statistical terms used in EBM. Training workshops should be set up to teach residents the skills needed to practice EBM.

## Supplementary Information

**Additional file 1.** Questionnaire on the Knowledge, attitude, and practice of evidence-based medicine among resident physicians in hospitals of Syria.

**Additional file 2.** EBM Syrian residents Quantitative dataset.

## Data Availability

The datasets generated and analyzed during this study are included in the supplementary files (Additional file 2).

## Abbreviations

EBM: Evidence-based medicine
KAP: Knowledge, attitude, and practice
Std. deviation: Standard deviation
